# Genome Sequence of the Probiotic Strain Bacillus velezensis Variant polyfermenticus GF423

**DOI:** 10.1128/MRA.01000-18

**Published:** 2018-09-13

**Authors:** Haeyoung Jeong, JungAe Kim, Soo-Keun Choi, Jae-Gu Pan

**Affiliations:** a Infectious Disease Research Center, Korea Research Institute of Bioscience and Biotechnology (KRIBB), Daejeon, Republic of Korea; Iowa State University

## Abstract

The genome sequence of the commercial probiotic strain “Bacillus polyfermenticus” GF423 was determined. Comparison of the 4.1-Mb genome sequence revealed Bacillus velezensis FZB42 as its closest relative. Based on the genome sequence, we propose that this probiotic strain be renamed Bacillus velezensis variant polyfermenticus.

## Abstract (precis)

The genome sequence of the commercial probiotic strain “Bacillus polyfermenticus” GF423 was determined. Comparison of the 4.1-Mb genome sequence revealed Bacillus velezensis FZB42 as its closest relative.

## ANNOUNCEMENT

Strains belonging to “Bacillus polyfermenticus” have long been used in Japan and the Republic of Korea for the treatment of intestinal disorders, and the immunomodulatory effects of these strains on human subjects have been demonstrated (1). At least two strains, SCD (KCCM 10104, also known as B. polyfermenticus n.sp. in the Korean Pharmacopoeia) (2, 3) and KJS-2 (4), were identified from their distinctive physiological properties. Strain SCD and its derivatives, such as KD21 (KFCC 11090) and KD33, are widely used for the development of enhanced probiotic products in academic research and in pharmaceutical companies in the Republic of Korea. Despite its importance in biomedical applications, Bacillus polyfermenticus is yet to appear in the Prokaryotic Nomenclature Up-to-Date database (https://www.dsmz.de/bacterial-diversity/prokaryotic-nomenclature-up-to-date), and genome-based analysis has not been performed for any of these strains.

Here, Bacillus polyfermenticus n.sp. was isolated from a Biscan G capsule (Binex Co., Ltd., Busan, Republic of Korea) and renamed GF423, and genome sequencing was carried out using the Illumina HiSeq 2000 platform. Bacterial culturing, library construction, and genome sequencing were carried out as described previously (5). A total of 2.23 Gb of reads (2 × 101 cycles) were trimmed using Trimmomatic version 0.32 (6) with SLIDINGWINDOW:4:20, MINLEN 75, and all other parameters being adopted from those used in the trimming section of A5-miseq version 20150522 (7), and 20-mer nucleotides below the abundance cutoff value of 50 were filtered out from the reads using khmer version 2.0 (8) to minimize contamination in the reads. *De novo* assembly using the CLC Genomics Workbench version 8.5.1 (Qiagen) with a word size of 64 yielded 33 contigs totaling 4,112,512 bp (47.95% G+C content). The *N*_50_ value and the largest contig length were 502,810 bp and 618,700 bp, respectively. Genome annotation was performed using the NCBI Prokaryotic Genome Annotation Pipeline (9) and the Rapid Annotation using Subsystem Technology (RAST) server (10).

We calculated overall genome-related indices using dRep version 2.0.5 (11) to investigate genomic relatedness to strains in the Bacillus subtilis species complex. The results revealed a tight cluster with strains belonging to the species Bacillus velezensis, with >98% genome-wide average nucleotide identity (gANI), including with the type strain NRRL B-41580 (GenBank accession no. LLZC00000000). Based on this result, we propose that B. polyfermenticus probiotic strains be renamed B. velezensis variant polyfermenticus. We suggest that “variant polyfermenticus” be added to reflect the history of strain usage in over-the-counter drugs as well as probiotics. In particular, the strain is most closely related to B. velezensis FZB42 (GenBank accession no. CP000560; 98.7% gANI and 89.0% digital DNA-DNA hybridization, the latter of which was calculated using Genome-to-Genome Distance Calculator version 2.1, available at http://ggdc.dsmz.de/distcalc2.php), which was formerly designated the type strain of B. amyloliquefaciens subsp. plantarum (12) but recently recognized as synonymous with B. velezensis (13, 14). Nucleotide sequences in GenBank, especially those from Bacillus polyfermenticus 16S ribosomal genes, should thus be reevaluated to avoid misleading strain identification.

One of the physiological characteristics of B. polyfermenticus that distinguishes it from B. subtilis is its capacity to produce acids from lactose but not from d-turanose (2), which was also observed from the type strain of B. velezensis (15). Additionally, this strain has the ability to ferment glucose to lactic acid (4). Our results emphasize the importance of genome sequence information for accurate species identification and elucidation of physiological characteristics of bacterial strains.

### Data availability.

This whole-genome shotgun project has been deposited in DDBJ/ENA/GenBank under accession no. QOHQ00000000. The version described in this paper is the first version, QOHQ01000000.
